# Cancer prevention programmes in Mexico: are we doing enough?

**DOI:** 10.3332/ecancer.2020.997

**Published:** 2020-01-06

**Authors:** Nicolás Padilla-Raygoza, Rebeca Monroy-Torres, Cuauhtémoc Sandoval-Salazar, Luz Elvia Vera-Becerra, María Esther Patiño-López, María de Lourdes García-Campos, Vicente Beltrán Campos, Mayra del Carmen Ortega Jiménez, Silvia del Carmen Delgado-Sandoval, Xóchitl Sofía Ramírez-Gómez, Sandra Neli Jimenez-García, Hilda Lissette López- Lemus

**Affiliations:** 1 School of Medicine, University of Celaya, Celaya CP38080, Mexico; 2 Laboratory of Nutrition and Safety Food, Department of Medicine and Nutrition, Division of Health Sciences, Campus Leon, University of Guanajuato, León CP 37670, Mexico; 3 Department of Nursing and Obstetrics, Division of Health Sciences, Campus Celaya-Salvatierra, University of Guanajuato, Celaya CP38110, Mexico; 4 Department of Medicine and Nutrition, Division of Health Sciences, Campus Leon, University of Guanajuato, León CP 37670, Mexico; 5 Department of Clinical Nursing, Division of Health Sciences, Campus Celaya-Salvatierra, University of Guanajuato, Celaya CP38110, Mexico

**Keywords:** cancer, prevention, public programmes, early diagnosis

## Abstract

Cancer has increased in all the countries of the world and Mexico is no exception. The recognised risk factors for the main types of cancer are reviewed and searched through the Mexican government web pages and cancer prevention programmes to tackle the risk factors in the population. The Mexican government, a member of the World Health Organization, shows that the main approach is an early diagnosis rather than prevention, forgetting that an ounce of prevention is better than a pound of cure. Effective public programmes should be promoted to reduce preventable risk factors in the population (smoking, nutrition, obesity, diet, environmental toxicity, sedentary lifestyle) and control the non-preventable factors (genetics) if we really want to control the incidence of different types of cancer.

‘An ounce of prevention is worth a pound of cure’ is applied to the health of men. bestliquidherbs.com

## Introduction

Cancer is a generic term that covers a group of illnesses characterised by the increase of abnormal cells that can invade adjacent body parts and spread to other organs [1]. The most common forms in men are: lung, prostate, colorectal, stomach and liver, while in women they are: breast, colorectal, lung, cervical and thyroid [1].

Cancer is the second cause of death in the world with 9.6 million deaths in 2018 [1]. The annual cost derived from cancer was 1.16 trillion dollars in 2010 [1].

It is believed that between 30% and 50% of cancers can be prevented by modifying risk factors such as reducing the consumption of tobacco products, consuming less alcohol, maintaining a healthy weight, increasing physical activity and avoiding risk factors relating to infections like hepatitis or human papillomavirus (HPV) [1, 2].

In Mexico, cancer is considered the third cause of death [3]. The most common types of cancer among Mexican men are: prostate, colorectal, testicular, lung and gastric. Among Mexican women, they are: breast, thyroid, cervical, uterine corpus and colorectal [4].

## Epidemiology of the main types of cancer in Mexico

### A third of cancer deaths are due to the following reasons

Using tobacco, which is the most important risk factor for cancer [5]. Hepatitis and HPV, which represents more than 25% of the cases of cancer in low- and middle-income countries [6].

Other factors to consider for cancer are an elevated body mass index (BMI), low ingestion of fruit and vegetables and a decrease in physical activity [2].

The Panamerican Health Organization (PHO) indicated that, in Mexico, in 2015, for every 100,000 inhabitants, prostate cancer caused the death of 13 males, while breast cancer caused the death of 11 women [7].

In its different forms, cancer has had sustained growth in the last 20 years, mainly due to an aging population, and changes in lifestyles and physical activity [8–10].

## Prevention from the World Health Organization’s (WHO) perspective

### The WHO promotes the prevention of cancer by trying to tackle the most common risk factors

Tobacco consumption, sedentary lifestyle, change in diet, use of alcohol, infections like hepatitis and HPV, environmental contamination, occupational carcinogens and radiation [7, 11].

The goal was a search for official preventive programmes on Mexican government agency websites to detect preventive programmes, not timely detection, of different types of cancers in Mexico.

The methodology was in general search: cancer prevention, Mexico government, smoking, obesity, diet, physical activity.

## Risk factors

### Smoking and cancer

Four thousand chemicals have been found in oral tobacco products, 60 of which are considered carcinogens; nicotine is the active drug in tobacco and there isn’t much information about the additives that are added into tobacco, and their effect on health. [12, 13].

Smoking is considered to cause 30% of all deaths due to cancer [12]. Carbon monoxide reduces the oxygenation of organs and tissues and nicotine can cause haemodynamic problems [13, 14].

Passive smoking is second-hand exposure to tobacco smoke; this smoke is made up of gases and particles like nicotine and various carcinogens and toxins [13, 15].

### Mexico adheres to the WHO for the control of tobacco and adopts the following actions

In Mexico in 2008, the general law was approved for the control of tobacco [16]; in 2009, in agreement with the General Law Regulation for control of tobacco, pictographs and warnings were implemented, which consisted of the fact that they should occupy 30% of the anterior side, 100% of the posterior side and 100% of one of the lateral sides, which is in line with the WHO guidelines [17]. Currently, a helpline number has been added to quit smoking if the user wants to. Also, as an additional measure, the sale of tobacco products to minors has been prohibited.

An increase in the price of tobacco products through taxes is the most effective measure to reduce its consumption [18]. It has been estimated that a 10% increase in the price has reduced the use of tobacco by 4% in high-income countries and by 8% in middle-income countries [19].

Creating smoke-free spaces, the total prohibition of advertising, sponsorship and promotion of tobacco products, combined with those already mentioned in the pictographs and warnings, as well as the tax increase on tobacco products, are the public policies to which the Mexican government has committed itself in the fight against tobacco use [18].

According to the National Survey on Drug Use and Health 2016–2017 (NSDUH 2016–2017), 17.6% of the Mexican population between 12 and 65 years old smokers, which represents 14.9 million smokers and of those 8.7% are women and 27.1% are men [20]; the places with the greatest exposure to second-hand tobacco smoke are bars (52.8%), restaurants (31.9%), public transport (26.0%), schools (25.4%) and work (15.3%) [19]; this shows that there is not enough vigilance by authorities, and people who do not denounce the violation of smoke-free spaces, or indifference of all parties involved.

An extremely important fact is that 98.4% of tobacco consumers know the health dangers of using tobacco [20]. Therefore, efforts aimed at reducing consumption should be designed to change the attitudes and motivations of smokers, because public policy efforts are not giving the expected results.

Other findings relevant to the inquiry are: the average age of starting to use tobacco products is 19.3 years old [19], although there are reports where this happens at an earlier age [21, 22].

91.8% of Mexicans support the law of 100% smoke-free spaces, and health warnings and pictographs achieved that 27.9% of smokers avoided consuming a cigarette in the last month [20].

## Nutrition and cancer

Cancer presents multiple risk factors, from genetics to environment; lifestyle is one of the associated environmental factors both in the prevention and in the denotation of a neoplasm [23, 24]. Diet forms part of this lifestyle. With the changes in quality of food, popularising industrialised foods, the population has been more exposed to different additives, preservatives, colorants, etc., which are incorporated as alternatives to increase the shelf life of foods, improve its image and consumer acceptance, but many of them are powerful carcinogens, which have been described once they have been launched on the market [25].

The increase in the consumption of processed foods combined with a low consumption of fruits and vegetables by part of the population shows the greatest exposure of various carcinogens and a low consumption of fiber and antioxidants like vitamin A, C, flavonoids, minerals like selenium, in addition to key amino acids (methionine) [26, 27].

Developing cancer will depend on the etiology and pathophysiology of the type of cancer, for example with gynaecological cancers, such as cervical cancer, whose main causative factor is the HPV, an oncogenic virus with different genetic varieties, it is known that development of dysplasia is greater when you have a diet that is low in antioxidants or lacks them entirely (vitamins and minerals such as folic acid) associated with smoking and other cofactors such as obesity. Therefore, maintaining a proper diet that is varied, complete, sufficient, adequate and balanced according to the conditions of each person is known to prevent the development of cancer [26–29].

Diets high in added sugars or those known for easy digestion such as high fructose corn syrup or sucrose, low fiber intake, saturated fat and sodium [24] have been implicated in it. In this sense, studies have been carried out in which the evidence shows that following a western dietary pattern may increase the risk of presenting some cervical neoplasia (OR = 3.26, 95% CI, 1.03–10.3, *p* < 0.05) [30]. Various studies have linked the benefits of various nutrients for their antioxidant role. A review in 2005 by García-Closas *et al* [31] included all the evidence found so far on the association that food has with the persistence of a cervical neoplasia [32–34]. *β*-carotene is the most important and frequent carotene in the diet but also *α*-carotene, lutein or lutein/zeaxanthin and beta-cryptoxanthin have probable protective effects for cervical cancer, but this has only been demonstrated in diet and not as an oral supplement [33, 34]. Vitamins A, C and E have been described as protectors against the development of pre-malignant lesions to cancer, but like carotene this effect has not been found with supplements or oral preparations [35–37]. The little, but well-founded evidence helps continue promoting the consumption of fruits and vegetables to help with the protective effect [38].

A study by Piyathilake *et al* [33] examined 315 cases of women with HPV, 16 diagnosed with NCI 2+ (90 participants) or NCI1 (225 participants), found a probability of presenting CIN 2+ in women with high level of folic acid (OR = 0.25, 95% CI, 0.10–0.58, *p* < 0.01 and OR = 0.40, 95% CI, 0.17–0.88, *p* = 0.02). On the other hand, Feng *et al* [39] found in 2338 women with high-grade NCI that consumption of vegetables with dark and light colours, onions, legumes, seeds, eggs and meat, reduced the risk of NCI with only the consumption of 1 weekly portion of vegetables (onions) (16.3), legumes (2.8), seeds (0.3–0.61), and meat (1.02), (OR = 0.58, 95% CI = 0.38–0.89, *p* = 0.011 ; OR = 0.59, 95% CI: 0.39–0.89; OR = 0.59, 95% CI = 0.39–0.88; and OR = 0.62 95% CI = 0.40–0.95) as well as [40] with consumption of fruits (<70 g/day) and vegetables (260 g/day) (OR = 2.84, 95% CI = 1.26–6.42, *p* = 0.06) [41, 42].

## Obesity and cancer

Cancer refers to various diseases that affect any cell group in the body, with the appearance and development of tumours or malignant neoplasms. Interestingly and as a main feature, it is observed that mitosis of cancer cells is rapid with an efficient ability to spread and invade adjacent tissues or organs (metastases). This last condition is the leading cause of death from cancer [1, 2].

Cancer is found worldwide as the second leading cause of death, and to a large extent, these deaths (70%) occur in low- and middle-income countries. Among the causes that favour the development of this disease are several factors which are mentioned below: ‘physical carcinogens, such as ultraviolet and ionising radiation; chemical carcinogens, such as asbestos, the components of tobacco smoke, aflatoxins and arsenic; and biological carcinogens, such as certain viruses, bacteria and parasites’. Similarly, aging is another fundamental factor in the onset of cancer, since in addition to the aforementioned factors there is a combination with the deficiency of cell regeneration and repair mechanisms given in the aging process [1, 2]. Similarly, alcohol consumption, poor diet and physical inactivity are the main risk factors for cancer in the world [1, 2]. In this section, those factors that are related to poor eating habits are of interest as well as a high BMI.

‘Obesity and overweight are defined as an abnormal or excessive accumulation of fat that can be harmful to health’ [42]. Being a complex and multifactorial condition, biological, genetic and environmental components are involved [43].

The relationship between obesity and risk for developing cancer is not something new and there is evidence that both genders with a high BMI have a higher risk of cancer development [44]. Studies indicate the association between BMI and cancer in various human organs and tissues [45], including endometrial, oesophageal, renal and pancreatic adenocarcinoma; hepatocellular carcinoma, gastric; meningioma; multiple myeloma; colorectal, breast, ovarian, vesicular and thyroid cancer [46].

The risk of developing cancer when obesity occurs may vary in different ethnic groups. For example, in a study conducted in the Hispanic and non-Hispanic population, it was found that there is an increased risk of breast cancer in non-Hispanic women with obesity compared to Hispanic women. However, more studies are needed to specifically assess the race variable and its mediating effect between obesity and the risk of developing cancer [47].

Among other factors that link obesity with cancer are sedentary lifestyles and nutrient poor diets or high in fat or sugar which produce oxidative stress [48], from the increase in reactive oxygen species and nitrogen. These are harmful not only to cells [49] by affecting lipids and proteins of systemic organs, but to the brain as well [50].

This relationship between obesity and cancer could be summarised at the cellular level from the cell group that accompanies adipocytes, which includes immune cells, stromal cells, blood vessels and neurons. Thus, within the functions of adipose tissue there is the storage of lipids and endocrine regulation of food intake with the release of leptin and fatty acid oxidation by adiponectin. In an obese patient, this tissue is affected, and inflammation is favoured with the release of cytokines IL-1, IL-6, TNF*α* and the transforming growth factor. The above results in the activation of monocytes and macrophages that release even more cytosines that can contribute to insulin resistance, maintaining inflammation and metabolic cell dysfunctions that affect the processes of cellular communication from cell membranes, to gene regulation in the cell nucleus [51].

It has also been postulated that the intestinal microbiota has an effect on obesity through the immune response and inflammation [52]. Additionally, it has been proposed that the microbiota plays an important role in establishing the pathophysiological conditions for cancer induced by said obesity [53].

Other hypotheses have recently been explored about the association between obesity and cancer development. This new evidence suggests that some particular components of the diet may have a greater role in increasing risk. For example, some proteases may decrease the function of cells that act as tumour suppressors. One of these is the chymotryptic subtilisin enzyme. Regarding the above, simple public health measures such as eliminating this enzyme in processed food procedures, which are highly consumed by people with obesity, could decrease the risk of proliferation and migration of some cells related to inflammation and oncogenesis [54]. When considering the above, the WHO proposes prevention strategies to address this health problem, in which it is indicated that ‘the modification or prevention of the main risk factors such as excess weight or obesity, poor diet in which insufficient amounts of fruits and vegetables are ingested and finally physical inactivity’ [1, 2].

In Mexico, ‘the prevention measures related to cancer are not enough or practically null and this may be due to the lack of knowledge on the part of the population, as well as insufficient equipment and infrastructure for health institutes and research centres’ [55] in this regard, only the national cancer control plan (NCCP) can be mentioned. Its objectives include strengthening cancer education, reducing incidence and mortality rates, developing a realistic prevention programme, guaranteeing cancer diagnosis and treatment based on best practices, and offering the best possible quality of life to patients and their families, in addition to promoting research in this area [56].

Regardless, there are programmes, strategies, regulations and guides to good practice for the prevention, control, and management of overweight and obesity in Mexico, some of which include the following, and which if well applied could contribute to the control of the obesity epidemic as well as to decreased incidences of cancer:

- The Programme of Specific Action for Prevention and Control of Obesity and Cardiovascular Risk 2013–2018, which prioritises prevention and health promotion actions considering successful practices based on scientific evidence to reduce the burden of chronic noncommunicable diseases in the Mexican population, including cost-benefit intervention strategies according to social determinants of health and with a gender approach [57].
- The National Strategy for the Prevention and Control of Overweight, Obesity and Diabetes was published in 2013 and promotes the development of a national public policy for the establishment of healthy dietary habits and physical activity in the community, involving public and private sectors, as well as a civil society [58].
- The Official Mexican Standard, OMS 008-SSA3-2017, for the comprehensive treatment of overweight and obesity which is mandatory for health professionals and establishments that provide services related to overweight and obesity. It establishes the characteristics for health professionals, the operating requirements that establishments must meet and the criteria for the comprehensive treatment of overweight and obesity [59].
- The Clinical Practice Guide for the diagnosis, treatment and prevention of overweight and obesity in adults, which makes recommendations based on scientific evidence to standardise national actions for evaluation, diagnosis, treatment and prevention of overweight and obesity in the first and second levels of care [60].

Finally, regarding this problem, the National Institute of Public Health (NIPH) published an article in their portal titled ‘Evidence for public policies for the control of obesity and chronic diseases’, whose objective was to compare evidence to substantiate public policies for the control of obesity and chronic illnesses generated by the NIPH and for other research groups [56].

Despite the above, prevention and control measures are not sufficient in Mexico, as this country has the second-highest prevalence of obesity in the adult population in the world [56], and is among the first places of childhood obesity worldwide [61] (www.unicef.org), making it one of the most important public health challenges due to its impact on the country’s productivity and economic development by increasing the direct and indirect costs of its treatment [56, 62].

## Cancer and genetics

Since cancer is a genetic disease, the cause, in addition to environmental factors, involves cellular changes at the genetic level that control how cells grow and divide; some of these changes cause cells to evade controls or to reproduce in disfigured ways. The changes can be somatic (acquired) or offspring (from germ cells) [63].

Inherited mutations play a major role in 5%–10% of all cancers and it is important to identify mutations. Various tumour markers are used, tests that can identify whether people have inherited the mutation without yet showing obvious signs of the disease. Such tests should be considered when evaluating the risk when there is a family history, noting that the inheritor of the mutation is predisposed to cancer if associated with other environmental factors [64].

A genetic test is one that looks for mutations in the deoxyribonucleic acid (DNA) that is associated with a greater probability of suffering some type of cancer, which can vary in terms of its level of expression (dominant, recessive, incomplete penetration, etc.) which explains why the symptoms vary from one person to another.

More than 50 hereditary cancer syndromes have been identified so far, most of them caused by dominant mutations. Among the most common is the breast and ovarian cancer syndrome associated with the BRCA1 genes, a gene located on chromosome 17, which helps suppress cell multiplication and BRCA2, with breast cancer being the most commonly diagnosed among Latino patients [65], is responsible for approximately 16% of cases of hereditary breast cancer [66], with mutations in the BRCA1 gene exon 9–13 being the most common among Hispanics [67]; TP53, which produces a tumour growth-inhibiting protein, is the most mutated gene in all cancers, and produces Li–Freumeni Syndrome, an inherited disease associated with an increased risk of cancer. In the case of the Mexican population, Next-generation sequencing (NGS) has improved the diagnosis of non-small cell lung cancer (NSCLC) and personalised treatment [68].

A systematic review of the literature found that in other countries such as the United Kingdom, the United States of America and Australia, 148 programmes have been identified to identify genetic factors associated with cancer, of which 66.45% were national programmes, 49.33% were regional or urban area programmes; as well as 64% being integrated into the health system, 32.21% were pilot programmes and only 5% were direct consumer services [69]; while in Mexico, there is a lack of specialists in genetics and genomic medical services [70].

It is recommended that candidates for genetic testing be those with a family history that indicates the presence of the suspected inherited mutation, considering that the test is of high predictive value, and that the information obtained helps provide early preventive health care [64].

Although part of the population in Mexico may have access to genetic analysis to identify genetic factors associated with the development of some types of cancer, in order to take preventive measures against environmental risk factors, for the early detection of a tumour or to take the option for chemotherapy or prophylactic surgery, which economically would be more affordable, these services are not in high demand in our country nor are there any government genetic counseling programmes to this day.

## Cancer and hormones

Breast cancer ranks second among cancer diagnoses worldwide and first to be linked as cancer linked to the female gender [71]. There are several factors associated with the development of the disease, of which lifestyle and environmental factors predominate up to 90%–95% [72]. Among these factors, those that increase morbidity and reactivation of mammary gland cancer, such as alcohol consumption, ionising radiation, and hormone therapy, among others, are of vital importance [73]. Other risk factors are age and genetic burden, mainly associated with a family history of breast cancer development, where the frequency of disease development may be as high as 80% [74].

The National Development Plan 2013–2018, in Mexico [75] establishes as a priority that the institutions of the National Health System and society as a whole ‘must make protection, promotion and prevention actions a priority axis for the improvement of health, in order to achieve the national goal of an inclusive Mexico’. It is also described in the presentation of the specific action programme for the prevention and control of cancer in women (PAEPCCM).

The PAEPCCM proposes prevention at three different levels that are carried out according to the specific population to which they are directed. The first actions are educational, aimed at the entire population, the second at women by age group, and the third for women who are at high risk [75].

The short- and medium-term outlook for Mexico in terms of breast cancer is not encouraging. The creation and implementation of prevention and timely detection programmes has proved insufficient to curb the occurrence of cases and deaths from these diseases [76].

Measures to reduce breast cancer mortality are based on screening and timely diagnosis through self-examination, clinical examination and mammography. Reviews of the beneficial or harmful effects of each of these interventions have changed detection policies.

These conclusions are based on the recent publication of two randomised trials involving about 400,000 women in Russia and Shanghai, comparing breast self-examination with doing nothing, mammography as a single screening intervention [77].

To avoid an increase in the incidence and prevalence of the disease, health systems have suggested two levels of prevention: primary prevention, which consists of eliminating the causes that lead to the onset of the disease and increasing or improving the immune system in the population, and secondary prevention, which aims to end the disease development process before its full symptoms are diagnosed, which can inhibit or prevent the development of a malignant tumour [78].

Over the last 2 decades, the emphasis has been placed on hormone-blocking therapy for cancers that show the oestrogen receptor (ER). This is achieved by cellular manipulation with anti-oestrogens, which compete with the ER in the breast tumour, or by systemic reduction of oestrogen levels with the use of luteinizing hormone agonists (LHRH) and in postmenopausal women with aromatase inhibitors (AI) that block oestrogen biosynthesis in non-ovarian tissues [79].

Blockade of oestrogen signs with endocrine therapies (tamoxifen or fulverstrant) is an effective treatment for breast cancer tumours that show oestrogen-*α* receptors. Unfortunately, the development of resistance to the oestrogen therapy has been reported as a frequent event that results in a relapse of the disease and a decrease in the patient’s overall survival [79, 80].

Understanding the difference between primary and secondary resistance as well as adjacent mechanisms is of vital importance to develop new research works and offer better prevention and treatment options. A significant proportion of patients have resistance again or primary or develop endocrine resistance during the course of treatment. Only 50% of patients with positive breast cancer for metastatic ER benefit from first-line endocrine therapy, and all the ones who initially respond acquire resistance, with relapse and growth of the tumour [81].

Also, with secondary resistance, progression-free survival (PFS) times with additional options of endocrine treatment are short [82].

Now genetic counselling and molecular diagnosis are routinely offered by family cancer clinics in specialised hospitals.

To those with positive results for a mutation, follow-up strategies and even chemoprevention and prophylactic surgery can be offered.

BRCA1 and BRCA2 are described as hereditary breast and ovarian cancer genes. The explanation of why changes in the function of BRCA1/2 have been associated precisely with breast cancer is because, during oestrogenic metabolism, highly reactive species capable of forming aberrant structures (adducts) in the DNA are generated. These products are found in greater quantities in those organs that respond strongly to oestrogen, such as the breast and the ovary. In patients with germinal mutations of BRCA1/2, the basic mechanisms for detection and repair of the damage are modified, which allows the accumulation of mutations associated with the formation of these adducts [83].

Now genetic counselling and molecular diagnosis are routinely offered by family cancer clinics in specialised hospitals.

To those with positive results for a mutation, follow-up strategies and even chemoprevention and prophylactic surgery can be offered [77].

## Physical activity and cancer

Cancer has multicausal origins. Some lifestyles, environmental work factors and social determinants play a key role in health or illness, so the best strategies to improve the health of the population are based on prevention and development of intersectoral health policies [84]. Prevention strategies are based on reducing risk factors and the early diagnosis of cancer [85].

There are three risk factors: tobacco, inadequate food and lack of physical activity, which contribute to the development of the most common chronic illnesses (cardiovascular disease, type 2 diabetes and cancer) which cause 50% of deaths in the world [84].

The WHO defines physical activity as any body movement produced by the skeletal muscles, with consequent energy consumption [85].

Regular physical activity at adequate levels reduces the risk of hypertension, coronary heart disease, stroke, diabetes, different types of cancer (breast and colon cancer) and depression [85].

Aiming to quantify the levels of physical activity in the adult population, the WHO created a global questionnaire on physical activity [global physical activity questionnaire (GPAQ)], which helps monitor insufficient physical activity as one of the main risk factors for developing noncommunicable diseases [85].

WHO and the American Cancer Society recommend that adults practice moderate physical activity at least 150 minutes a week, or intense physical activity at least 75 minutes a week, or an equivalent combination between moderate and intense activity [85, 86]. It has been showed that this level of physical activity has clear health benefits, including the decrease in the rates of premature death and in the incidence of mortality due to several types of cancers [86].

Physical activity can reduce the risk of several types of cancer, such as breast, colon and endometrial as well as advanced prostate cancer and possibly pancreatic cancer [86].

Regular physical activity helps maintain a healthy body weight by balancing caloric intake with energy expenditure, and it can help prevent certain kinds of cancer through direct and indirect effects, including the regulation of sex hormones, insulin and prostaglandins, and having several beneficial effects on the immune system [86].

In one descriptive and retrospective study with 22 patients with colorectal cancer, the GPAQ-WHO was applied, finding that moderate and intense physical activity was between 418 and 475 minutes per week, respectively, both higher than the recommendation (*p* = 0.029, *p* = 0.015) [87]. The subjects of this research exceeded what was recommended because most of them were agricultural workers from rural areas of the region [87].

Hardikar *et al* [88] showed that people who were physically active before the diagnosis of colorectal cancer experienced a better survival rate than those who were inactive or minimally active. Similarly, another study showed that physical activity performed before or after the diagnosis of cancer is related to the decrease in the risk of mortality among breast and colorectal cancer survivors [89].

Numerous epidemiological studies have shown that regular physical activity reduces the risk from 10% to 30% of developing colon, breast and endometrial cancer in postmenopausal women and possibly premenopausal breast, prostate, lung and pancreatic cancer [90]. Around 9%–19% of the most frequent types of cancer can be attributed to the lack of physical activity [90].

The study by Aguilar Cordero *et al* [91] found that physical exercise turned out to be a protective factor against breast cancer. In addition, there was a direct relationship between breast cancer in women who didn’t perform any kind of physical activity and those in the control group where all women practiced some kind of physical exercise [91].

Another study recruited participants over the age of 18 between the years of 2006 and 2014, finding that, in women, physical activity was inversely associated with the risks of colorectal, uterine and breast cancer [92]. The highest level of physical activity was associated with a reduction of risks of colorectal cancer (OR = 0.60; IC: 0.44–0.84) and uterine cancer (OR = 0.47; IC: 0.27–0.80) [91]. Reduced risks of breast cancer were associated with low levels (OR = 0.66; IC: 0.51–0.86) and moderate levels (OR = 0.72; IC: 0.57–0.91) of physical activity [92]. Finally, it was shown that there was no association between the levels of physical activity and the risk of cancer for men [92].

## Environmental toxins, foods and cancer

As mentioned earlier, there are different factors that influence the development of cancer in men, and to date, there have been numerous research works on the effect that some components of industrialised foods and preparation techniques such as charcoal roasting have in contributing to the development of cancer [93].

In this regard, in several *in vitro* and *in vivo* toxicological studies, the carcinogenic and mutagenic effects that produce different additives present in industrially processed foods such as preservatives, dyes, sweeteners, flavourings, etc., have been evaluated. These compounds are present in a very wide range of foods such as drinks, pastries, processed meats, sausages and preserves, to name a few [94, 95].

In Mexico, the Federal Commission for Protection against Health Risks (COFEPRIS in Spanish) as well as the Food and Drug Administration (FDA) in the United States, ensure that substances incorporated as additives in the manufacture of industrialised foods be safe for human consumption [96, 97, 4, 5]. It is important to note that, although it has been shown in studies with cell cultures and in laboratory animals that some food additives and preservatives can be mutagenic and carcinogenic at certain concentrations and at different exposure times, there is no conclusive scientific evidence showing that this effect is seen in human beings exposed daily or occasionally to the concentrations that are present in this type of food [94, 95, 2, 3]. This is probably why they continue to be significantly produced, distributed and consumed in developed and developing countries.

In other words, the act of exposing oneself to a dangerous substance does not necessarily pose a risk for developing cancer. Various factors are required, such as an individual’s general state of health and lifestyle, the amount of the toxic substance ingested and the time spent consuming said substance [98, 6].

However, the picture is different if we talk about cancer prevention since these results are very important, because they at least allow us to identify substances that are dangerous and also represent a risk as carcinogens [98, 6]. Consequently, their consumption should be small or completely avoided in a diet.

Although at a global level there is a lot of information with respect to this, there are no public policies, strategies, or national programmes in Mexico that raise awareness, limit or diminish the consumption of processed foods specifically to prevent the development of cancer.

In this regard, Mexico relies on the National Strategy for Prevention and Control of Overweight, Obesity and Diabetes. The principal focus is on reducing the prevalence of obesity, labelling foods to show the calories they contain, and reducing the consumption of sugary drinks such as soda. On the other hand, the tax payment law on sugary drinks and foods of high caloric density was implemented, with the purpose of raising the final cost of these products for the consumer and in doing so reduce their consumption [99,100, 7, 8]. However, whether these measures have had an impact on reducing the consumption of sugary drinks and the prevalence of obesity and chronic degenerative illnesses like diabetes in Mexico has yet to be evaluated.

Even though there are no public policies in Mexico for reducing the consumption of processed foods, various Official Mexican Standards can be found in the Official Journal of the Federation, such as the Official Mexican Standard NOM-251-SSA1-2009, hygiene practices for the processing of foods, drinks or dietary supplements; Official Mexican Standard NOM-130-SSA1-1995, goods and services; food kept in hermetically sealed containers and subjected to thermal treatment; Health provisions and specifications; Official Mexican Standard NOM-213-SSA1-2002, products and services; processed meat products; sanitary specifications; methods of testing; Mexican Official Standard NOM-242-SSA1-2009, products and services; fresh, refrigerated, frozen, and processed fishery products; sanitary specifications and methods of testing and the Official Mexican Standard NOM-127-SSA1-1994; environmental health; water for human consumption and use- permissible limits of quality and treatment to which water must be subjected for its potability. To name a few, it is the requirement for producers of processed foods to guarantee chemical and microbiological safety with respect to the permissible limits of heavy metals and various additives to guarantee safety in its consumption [101–105, 9–13].

On the other hand, there are public policies, national programmes and official Mexican regulations directed towards reducing the sale and consumption of alcohol, more so for the effects this substance has on the Central Nervous System than to prevent the development of cancer. Here, it is important to indicate that the excessive consumption of alcohol has been explained as a risk factor for developing cancer of the oral cavity, pharynx, larynx, oesophagus, liver, colon, rectum, and breast. However, governmental initiatives to reduce the consumption of this substance does not include this aspect [106–107, 14–15].

As shown earlier, Mexico has no public policies for reducing the consumption of processed foods that are considered to pose risks for developing cancer. However, there are indeed national programmes that favour the adoption of healthy lifestyles, of which one of them is to eat a healthy and balanced diet that recommends a greater consumption of fruits and vegetables, less consumption of red meats, sugary drinks, salt and processed foods, all with the objective of preventing the development of non-transmissible diseases such as obesity, diabetes, cardiovascular diseases and cancer.

With respect to main environmental toxins identified as carcinogenic, these are arsenic, hexavalent chromium, cadmium, asbestos, radiation and various organic compounds used in diverse industrial sectors.

Regarding heavy metals, arsenic is considered to be a carcinogenic agent, and the main source of exposure is water consumed by humans. In this regard, the metal can also enter through foods such as fruits and vegetables that have been washed with water contaminated with the metal, and on the other hand, through the consumption of milk and meat from animals fed with fodder or grains contaminated with arsenic [108, 16]. This exposes the big problem of how these toxins are incorporated into the food chain, causing a phenomenon of bioaccumulation and biomagnification so that the final amount to which a human is exposed by consumption of food and water contaminated with the metal is greater, and with them, there would be a greater risk of developing cancer due to arsenic [98, 6]. Prolonged exposure to high concentrations of arsenic is linked to a greater risk for cancer of the bladder, skin, lung, digestive pathways, liver, kidney and lymphatic and hematopoietic cancers [98, 109, 110, 6, 17, 18].

In regard to reducing exposure to heavy metals and providing potable water, Mexico has the Public Policy for Improving the Efficiency of Urban Systems of Potable Water and Sanitation in Mexico (National Water Commission). This vision was fully aligned with the National Water Program 2007–2012, which establishes the vision of ‘being a nation that has a sufficient quantity and quality of water, that recognises its strategic value, uses it in an efficient way, and protects bodies of water in order to guarantee sustainable development and to preserve the environment.’ Currently, the objective of the National Water Program 2014–2018 is to achieve water security and sustainability in Mexico [111, 112, 19, 20].

In order to comply with the above, Mexico has several Official Mexican Standards, NOM-127-SSA1-1994, environmental health, water for human use and consumption-permissible limits of quality and treatments to which water should be subjected for purification [105, 13]. This standard specifies the physical, chemical, and organoleptic characteristics, such as the permissible limits of bacteriological and radioactive characteristics, as well as chemical constituents such as heavy metals, trace elements, pesticides, and organic solvents that water must meet in order to be considered safe with respect to its use and consumption.

On the other hand, hexavalent chromium has many applications such as in tanneries, ceramics, welding, and chrome painting industries. This metal enters the body either through the inhalation of dust and vapours or through direct contact with the skin and eyes. Occupational exposure to this metal is linked to the development of cancer of the nasal cavity, the paranasal sinus, and the lung [98, 6].

However, cadmium is a metal which is found in the earth’s crust and is mainly used for manufacturing nickel-cadmium batteries (NiCd), the production of ceramics, and is also found in cigarettes. Exposure to this metal is linked to the development of lung cancer. Consequently, tobacco use increases the risk of suffering from cancer if the individual is also exposed occupationally to cadmium [98, 6].

With respect to chromium and cadmium, there are no public policies oriented towards reducing the use of these metals in various industrial sectors. There are only Official Mexican Standards which monitor industrial waste utilising these metals to make sure they don’t contaminate water and mud, as well as permissible limit values in products used by humans. For that purpose, chromium is dealt with by the NMX-AA-044-SCFI-2014 Water Analysis – measuring hexavalent chromium in natural, salt, sewage and treated sewage water – method of testing; and for cadmium with the Official Mexican Standard NOM-231-SSA1-2002, articles of glazed pottery, glazed ceramics and porcelain. Limits for lead and soluble cadmium. Method of testing. NOM-212-SCFI-2016, batteries and primary cells, maximum permissible limits for mercury and cadmium, specifications, methods of testing and labelling. Likewise, the Official Mexican Standard NOM-047-SSA1-2011, Environmental health-biological markers of exposure for personnel exposed occupationally to chemical substances [113–116, 21–24], is applied to diminish the risk of exposure to both metals.

Unlike arsenic, chromium and cadmium, asbestos or amianthus are amorphous fibrous mineral and are used in the automotive industry for the production of brake pads and brakes, as well as in the production of sewage pipelines and waterproof roof coatings [98, 6]. Exposure to this metal mainly affects the development of lung cancer but also laryngeal and ovarian cancer [117, 25]. Although this metal is considered a carcinogen and its use is prohibited in other countries, it is still used in Mexico and therefore poses a risk for the development of cancer in workers exposed occupationally and in families who use products developed with asbestos. In particular, the Official Mexican Standard NOM-125-SSA1-1994. This establishes the sanitary requirements for the process and the use of asbestos [118, 26]. However, with scientific and clinical evidence on the risk of development of lung cancer due to exposure to asbestos, it is urgent that the Ministry of Health bans the use of asbestos in Mexico.

Another environmental contaminant with potential carcinogenic risk is ionising radiation such as x-rays, gamma rays, radon, alpha and beta particles, to mention a few, and they can cause leukaemia, lung and thyroid cancer. It has been observed that the younger the person is who is exposed to this kind of radiation, the greater the risk [119, 27]. In the case of X-rays and gamma rays, exposure is recommended only if the expected medical benefit is greater than the risk to the patient. The Official Mexican Standard NOM-012-STPS-2012 specifies the Safety and Health Conditions at workplaces where sources of ionising radiation are handled, and the objective of Official Mexican Standard NOM-008-NUCL-2011, Control of radioactive contamination, is to specify the criteria under which controls should be established to minimise the exposure of personnel occupationally exposed to surface radioactive contamination and airborne radioactive contamination [120, 121, 28, 29].

Polychlorinated biphenyls (PCBs) are aromatic compounds containing one to ten chlorine atoms attached to a biphenyl nucleus. They are used in electrical equipment (capacitors, transformers and ballasts). In the 1980s, their use was banned worldwide because they have a negative impact on the environment and because they are carcinogenic [122, 30]. Official Mexican Standard NOM-133-SEMARNAT-2015, Environmental Protection-PCBs-Management Specifications, establishes specifications for the environmentally sound handling and disposal of hazardous waste containing or contaminated with PCBs, involving the removal of PCBs, as well as for the handling and treatment of PCB equipment [123, 31].

Dioxins and Furans are compounds formed as byproducts of incineration from the production of chlorinated herbicides. The most toxic dioxin is the congener 2,3,7,8-tetrachlorodibenzo-p-dioxin and animal studies show that it is carcinogenic [124, 125, 32, 33]. To reduce exposure to these substances, Mexico has the Official Mexican Standard NOM-166-SEMARNAT-2014, which establishes the maximum permissible limits for the emission into the atmosphere of lead, total hydrocarbons, nitrogen oxides and dioxins and furans from secondary lead smelting processes, including the corresponding test methods and operating specifications [126, 34].

Polycyclic aromatic hydrocarbons are organic compounds formed mainly by the incomplete combustion of organic matter and in various industrial processes. Benzopyrene belongs to this group and is formed as a product of the combustion of cigarettes and charcoal for cooking [127, 35]. Smokers and people who frequently consume barbecued foods have greater exposure to these compounds, which are a greater risk factor in the development of cancer of the lung, oral cavity, stomach, colon and rectum. To avoid exposure, it is suggested not to smoke actively or passively, as well as not to use barbecuing as a food preparation technique. These compounds may also be present in foods processed by smoking [93, 1].

Finally, regarding environmental toxins emitted into the atmosphere, it is important to note that Mexico has a National Strategy for Air Quality 2017–2030. The goal of this policy is to improve air quality in order to prevent health problems in the population and conserving ecosystems [128, 36].

On the other hand, there are also management programmes to improve air quality (ProAire) and the National Inventory of Pollutant Emissions Criteria (INEM), which report emissions of pollutants from industry, transportation, commerce, services, homes, vegetation and soil generated in the country [129, 130, 37, 38]. Nevertheless, these programmes do not have specific actions for the prevention of cancer caused by toxic environmental carcinogens. There are only official Mexican standards to minimise the emission of pollutants and exposure to the environmental toxins mentioned in this section [105, 131–134, 13, 39–42].

## Conclusions

Although in the scientific literature and in the political discourses on health much is spoken of prevention, the reality is that the evidence shows weakness in Mexico and in many countries about the programmes designed for the prevention of cancer. If we review the prevention levels of Leavell and Clark, prevention includes health education and health promotion, as well as specific protection.

Decision-makers should focus on more successful cancer prevention programmes in the areas outlined by the WHO: smoking, obesity, nutrition, food, environmental toxins, genetic detection of cancer-promoting genes, increasing physical activity; if the population acquired a healthy lifestyle in these areas, the frequency of cancer would fall.

If we manage to motivate the population not to consume tobacco products, to maintain a healthy diet, to maintain a BMI at a suitable level, you would see the incidence of the different types of cancer going down.

In order to take advantage of the advances that science offers us to identify the presence of cancer at an early stage, it is important to make the population aware of its use, and that appropriate preventive measures can be taken, which in the long run may be less expensive than radiotherapy or chemotherapy.

So far, in our country, the measures established in the official guidelines target the timely prevention of breast cancer by carrying out appropriate detection and treatment, working at the secondary prevention level. However, there is no public policy that allows primary prevention to reduce the incidence or development of breast cancer or any other type of cancer, perhaps because there are multiple factors that contribute to its development and the cost of prevention could become greater than its secondary prevention. So, it is still essential to understand how lifestyle and additional factors influence the development of the disease, and to be able to establish the mechanism of endocrine resistance in order to develop therapeutic agents that can reverse it or avoid its development.

Most of the budget is focused on timely detection, which is not prevention. We must strive to establish prevention programmes that really motivate the population to a real change in lifestyle, and then we can foresee a decrease in the frequency of cancer in the Mexican population.

## Conflicts of interest

All authors declare that there are no conflicts of interest.

## Funding statement

The authors did not receive any funding for this review.

## Authors’ contributions

Nicolás Padilla-Raygoza designed the review, wrote the sections: introduction, epidemiology of major types of cancer in Mexico, prevention from the perspective of the WHO, smoking and cancer, conclusions and arranged the entire manuscript.

Rebeca Monroy-Torres: wrote the section on nutrition and cancer and the conclusions

Cuauhtémoc Sandoval-Salazar wrote the section on obesity and cancer, and conclusions.

Luz Elvia Vera-Becerra: collaborated in the writing of the section on obesity and cancer, and conclusions.

María Esther Patiño-López collaborated in the writing of the section on obesity and cancer, and conclusions.

María de Lourdes García-Campos collaborated in the writing of the section smoking and cancer, and conclusions.

Vicente Beltrán Campos wrote the section on hormones and cancer, and conclusions.

Mayra del Carmen Ortega Jiménez wrote the section on hormones and cancer, and conclusions.

Silvia del Carmen Delgado-Sandoval wrote the section on genetics and cancer, and conclusions.

Xóchitl Sofía Ramírez-Gómez wrote the section on food, environmental toxins and cancer, and conclusions.

Sandra Neli Jimenez-García: collaborated in the section on foods, environmental toxins and cancer, and conclusions.

Hilda Lissette López- Lemus wrote the section on physical activity and cancer, and conclusions.
